# Establishment and application of cross-priming isothermal amplification coupled with lateral flow dipstick (CPA-LFD) for rapid and specific detection of red-spotted grouper nervous necrosis virus

**DOI:** 10.1186/s12985-015-0374-5

**Published:** 2015-09-26

**Authors:** Zi Dan Su, Cheng Yin Shi, Jie Huang, Gui Ming Shen, Jin Li, Sheng Qiang Wang, Chao Fan

**Affiliations:** Key Laboratory of Sustainable Development of Marine Fisheries, Ministry of Agriculture, P. R. China, Yellow Sea Fisheries Research Institute, Chinese Academy of Fishery Sciences (CAFS), Qingdao, P. R. China; Function Laboratory for Marine Fisheries Science and Food Production Processes, Qingdao National Laboratory for Marine Science and Technology, Qingdao, P. R. China; College of Fisheries and Life Science, Shanghai Ocean University, Shanghai, P. R. China

**Keywords:** Red-spotted grouper nervous necrosis virus (RGNNV), Cross-priming isothermal amplification (CPA) system, Lateral flow dipstick (LFD), Detection

## Abstract

**Background:**

Red-spotted grouper nervous necrosis virus (RGNNV) is an important pathogen that causes diseases in many species of fish in marine aquaculture. The larvae and juveniles are more easily infected by RGNNV and the cumulative mortality is as high as 100 % after being infected with RGNNV. This virus imposes a serious threat to aquaculture of grouper fry. This study aimed to establish a simple, accurate and highly sensitive method for rapid detection of RGNNV on the spot.

**Methods:**

In this study, the primers specifically targeting RGNNV were designed and cross-priming isothermal amplification (CPA) system was established. The product amplified by CPA was detected through visualization with lateral flow dipstick (LFD). Three important parameters, including the amplification temperature, the concentration of dNTPs and the concentration of Mg^2+^ for the CPA system, were optimized. The sensitivity and specificity of this method for RGNNV were tested and compared with those of the conventional RT-PCR and real-time quantitative RT-PCR (qRT-PCR).

**Results:**

The optimized conditions for the CPA amplification system were determined as follows: the optimal amplification temperature, the optimized concentration of dNTPs and the concentration for Mg^2+^ were 69 °C, 1.2 mmol/L and 5 mmol/L, respectively. The lowest limit of detection (LLOD) of this method for RGNNV was 10^1^ copies/μL of RNA sample, which was 10 times lower than that of conventional RT-PCR and comparable to that of RT-qPCR. This method was specific for RGNNV in combination with SJNNV and had no cross-reactions with 8 types of virus and bacterial strains tested. This method was successfully applied to detect RGNNV in fish samples.

**Conclusions:**

This study established a CPA-LFD method for detection of RGNNV. This method is simple and rapid with high sensitivity and good specificity and can be widely applied for rapid detection of this virus on the spot.

## Background

Nervous necrosis virus (NNV) is a single-stranded RNA virus, belonging to the genus *Betanodavirus* within the family *Nodaviridae* whose genome consists of two molecules of RNA: RNA1 and RNA2. This virus is the pathogen that causes the viral nervous necrosis (VNN) in many species of fish. It can lead to large-scale death of the larvae and juveniles and the cumulative mortality can be as high as 100 % within 1 week after infection [1]. This virus is widely distributed. There have been reports about its epidemic in Asia, Australia, North America, and Europe [2, 3]. NNVs are divided into four genotypes, including barfin flounder nervous necrosis virus (BFNNV), red-spotted grouper nervous necrosis virus (RGNNV), striped jack nervous necrosis virus (SJNNV) and tiger puffer nervous necrosis virus (TPNNV). Among them, RGNNV is the most popular one. Currently, there have been no methods available for effective treatment of VNN caused by these types of virus. Thus, it is of extreme importance to establish a rapid and highly sensitive method for detection of these pathogens for prevention and control of this disease.

Currently, there have been several methods available for detection of NNV, including enzyme-linked immunosorbent assay (ELISA) [4], indirect fluorescent antibody test (IFAT) [5], cell culture [6] and molecular biology-based methodologies [7–13]. Among them, the molecular biology-based methodologies are more rapid and accurate and thus, the most widely used methods applied in the detection of NNV in fish samples. The molecular biology-based methodologies include the conventional reverse transcription-polymerase chain reaction (RT-PCR) [7, 8], loop-mediated isothermal amplification (LAMP) [9–11], real-time quantitative RT-PCR (qRT-PCR) [12, 13] etc. While these methodologies have been used in detection of NNV, each of them has certain limitations and shortcomings. For instance, the sensitivity for conventional RT-PCR is not high enough as its lowest limit of detection (LLOD) is as high as 10^2^ copies and thus, it does not meet the requirement for prevention and control of NNV. Although qRT-PCR is highly sensitive, this method requires the expensive equipment and reagents and can’t be used on the spot. While LAMP is relatively sensitive and can be used on the spot, its specificity was relatively low due to the use of staining dyes for color development of the amplified products. Cross-priming isothermal amplification (CPA) is a currently developed, nucleic acid-based amplification method which is rapid and highly effective [14]. With this method, at least one cross primer is used, making the sequences of the amplified product to form semi-loop single-stranded structure or branched structures. Under the action of *Bst* DNA polymerase which possesses the strand displacement activity, the targeted DNA sequence is continually amplified at a constant temperature. This technology has been successfully applied in the detection of many types of bacteria and virus, including *Mycobacterium tuberculosis* [15], *Enterobacter sakazakii* [16], *Acidovorax citrulli* [17], fowl adenovirus [18], and white spot syndrome virus [19] etc. In term of the analysis of nucleic acid-based amplified products, lateral flow dipstick (LFD) detection technology has been widely applied because it has a characteristic of rapid reaction and can be easily carried [20–22]. The principle of LFD is as follow: the amplified nucleic acid product doubly labeled with FITC and Biotin is drop-wise added on the dipstick, which interacts with the anti-biotin antibody- carrying colloidal gold and this complex is migrated with the liquid flow along the dipstick to the detection line where it is captured by anti-FITC antibody and aggregated, finally forming a red and macroscopical strip on the detection line. CPA coupled with LFD (CPA-LFD) may allow achieving the rapid and effective detection of pathogens on the spot [20, 23].

In this study, we established a cross-priming-isothermal amplification coupled with lateral flow dipstick method for specific detection of RGNNV and applied this method in detection of RGNNV in fish samples. The establishment of this method has provided a convenient way for rapid detection of RGNNV on the spot for those laboratories that have only simple equipment or aquaculture farms.

## Methods

### Fish Samples and RNA isolation

Moribund half-smooth tongue sole (*Cynoglossus semilaevis*) juveniles of 35 days post-hatching were collected from Shandong province, China, in 2012. These RGNNV-infected juveniles were confirmed with the RT-PCR detection method recommended by World Organization for Animal Health (OIE). Total RNA was isolated from the head (30 mg) of juveniles by using Quickgene RNA Tissue Kit (KURABO, Japan) according to the procedures provided by the instructional manual. The concentration of total RNA was measured and the total RNA samples were stored at −80 °C for subsequent use and analysis.

### Primers design of CPA-LFD

**Fig. 1:**
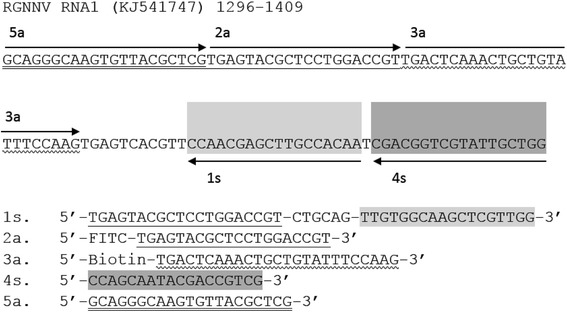
Locations and sequences of primers. The nucleic acid sequence of the targeted conserved region (1296–1409) of RGNNV RNA1 (KJ541747). CTGCAG is the restrict digestion site of *Pst*I, the 5’-ends of primers 2a and 3a were labeled with FITC and Biotin, respectively

Based on the sequence in the conserved region of RGNNV RNA1 (GenBank accession no. KJ541747), five CPA-LFD primers (1 s, 2a, 3a, 4 s, 5a) were designed with the on-line Primer Explorer V4 software (http://primerexplorer.jp/elamp4.0.0/index.html). The *Pst*I restrict digestion site, CTGCAG, was included into the primer 1 s. The primers were synthesized by Sangon Biotech (Shanghai) Co., Ltd. (Shanghai, China). The detailed sequences of the targeted DNA regions and their locations were shown in Fig. 1.

### Preparation of RGNNV RNA1 standard

In order to obtain stable and uniform virus RNA as the positive control template, the DNA fragments of RGNNV RNA1 gene were cloned according to the method described by Mu et al. [7]. The cloned DNA fragments were *in vitro* transcribed to prepare the RNA standard samples. The major procedures were as follows: the cDNA for RGNNV RNA1 was synthesized with TransScript First-Strand cDNA Synthesis SuperMix (TransGen, China). A 1032 bp of cDNA fragment (from 1033 bp to 2064 bp of RGNNV RNA1, KJ541747) was amplified via PCR and cloned into transcriptional vector pMD20 (TaKaRa, China). The resulting recombinant plasmid was transfected into competent *Escherichia coli* DH5α cells for amplification. After being purified, the amplified plasmid DNA was digested with *EcoR*I to linearize it and the *in vitro* transcription was conducted according to the procedures described in the operational manual of SP6 RNA Polymerase (TaKaRa, China). The transcribed products were firstly treated with DNase I, extracted with phenol/chloroform, dissolved in DEPC-treated water, and used as the RGNNV RNA1 standard. Before use, the extracted RNA1 standard was checked for integrity by RNA gel electrophoresis. The concentration was measured with Nanodrop and its copy number was calculated.

### Establishment of CPA-LFD for RGNNV and optimization of the amplification conditions

The total volume of the initial CPA-LFD amplification system was 25 μL, including 4 mmol/L MgCl_2_, 1.2 mol/L Betaine (Sigma-Aldrich), 1.0 mmol/L dNTPs (Takara, China), 1.6 μmol/L primer 1 s, 0.8 μmol/L primers 2a/3a, 0.2 μmol/L primers 4 s/5a, 2.5 μL of 10 × ThermoPol Buffer, 8 units (U) of *Bst* DNA polymerase (Warmstart 2.0, New England Biolabs, China), 28 U of M-MLV reverse transcriptase (Takara, China) and 1 μL of 10^5^ copies/μL RGNNV RNA1 standard. The reaction system was settled in a metal water bath and incubated at 63 °C for 60 min. The reaction was terminated by incubation at 80 °C for 5 min. Six (6) μL of the reaction product was taken out and drop-wide loaded onto the flow dipstick (Ustar, China) and the results were observed.

In order to find out the optimal amplification conditions, in this study, the amplification temperature, the concentration of dNTPs and concentration of Mg^2+^ were optimized successively. That means each optimization test uses previously optimized parameters as initial protocol [19]. For optimization of the amplification temperature, the amplification temperatures were set at 61, 63, 65, 67, 69 and 71 °C, respectively; For optimization of the concentrations of dNTPs, the dNTPs in the reaction system were set at 0.6, 0.8, 1.0, 1.2, 1.6 and 2.0 mmol/L, respectively; For optimization of the Mg^2+^ concentration, the concentrations of Mg^2+^ in the reaction system were set at 3, 4, 5, 6, 7 and 8 mmol/L, respectively.

### Comparisons in sensitivities among CPA-LFD, conventional RT-PCR and qRT-PCR for RGNNV detection

In order to test and compare the sensitivities of CPA-LFD, conventional RT-PCR and qRT-PCR detection methods, 1 μL of RGNNV RNA1 standard sample solution was taken out and a 10-fold series dilution within the range of 10^6^–10^0^ copies/μL was made and used as the template. They were amplified with CPA-LFD, conventional RT-PCR, and qRT-PCR, respectively. Among which, the amplification with CPA-LFD was conducted under the optimized conditions described above. The conventional RT-PCR and qRT-PCR were conducted according to the methods described by Dalla Valle et al. [13] with the primers sets of Q-RdRP-1 and Q-RdRP-2.

### The specificity of CPA-LFD for RGNNV detection

Specificity test for CPA-LFD detection method was conducted with nucleic acid prepared from 9 types of the common pathogens present in fish as the templates. They included red-spotted grouper nervous necrosis virus (RGNNV), viral hemorrhagic septicemia virus (VHSV), infectious pancreatic necrosis virus (IPNV), turbot reddish body iridovirus (TRBIV), lymphocystis disease virus (LCDV), infectious salmon anaemia virus (ISAV), *Vibrio harveyi, V. anguillarum* and *Aeromonas hydrophila*. The nucleic acid prepared from the head of healthy juveniles of *C. semilaevis* was used as the negative control. Specificity test of CPA-LFD detection method was conducted under the obtained optimal system.

Because it is difficult to obtain virus strains, the specificity test for CPA-LFD detection method didn’t include other three genotypes of NNVs (i.e. BFNNV, SJNNV and TPNNV). As a remedy, the sequence alignments between our designed primers and the type species of four NNV genotypes (GenBank accession no. NC_011063 for BFNNV, NC_008040 for RGNNV, NC_003448 for SJNNV and NC_013640 for TPNNV) were performed by BLAST on NCBI website.

### Application of the CPA-LFD for RGNNV detection in fish samples

Nine (9) samples of *C. semilaevis* juveniles whose infection status was unknown were tested for RGNNV with the established CPA-LFD detection method, LAMP-based Highly Sensitive and Rapid Detection Kit for RGNNV (Yellow Fisheries Research Institute, China) and qRT-PCR described above, respectively. The detected results with the three methods were compared. The major procedures for detection with LAMP kit were as follows: approximately 0.1 g of the head tissue sample of *C. semilaevis* was taken, grounded into homogenate within the sampling tube; the pasty homogenate samples were dipped with toothpick and dropped on the sampling membrane to make it fully wetted. Several drops of the rapidly dried liquid were dropped onto the sampling membrane, which was then transferred to the washing tube and vigorously shaken for 3–5 min. The sampling membrane was transferred to the amplification detection tube, which was settled in water bath and incubated at 63 °C for 60 min. The amplification detection tube was then heated at 95 °C, evenly mixing the dye sealed on the cap of the tube and the reaction mixture. The changes in color were observed by eyes.

## Results

### The establishment and optimization of CPA-LFD amplification system

**Fig. 2:**
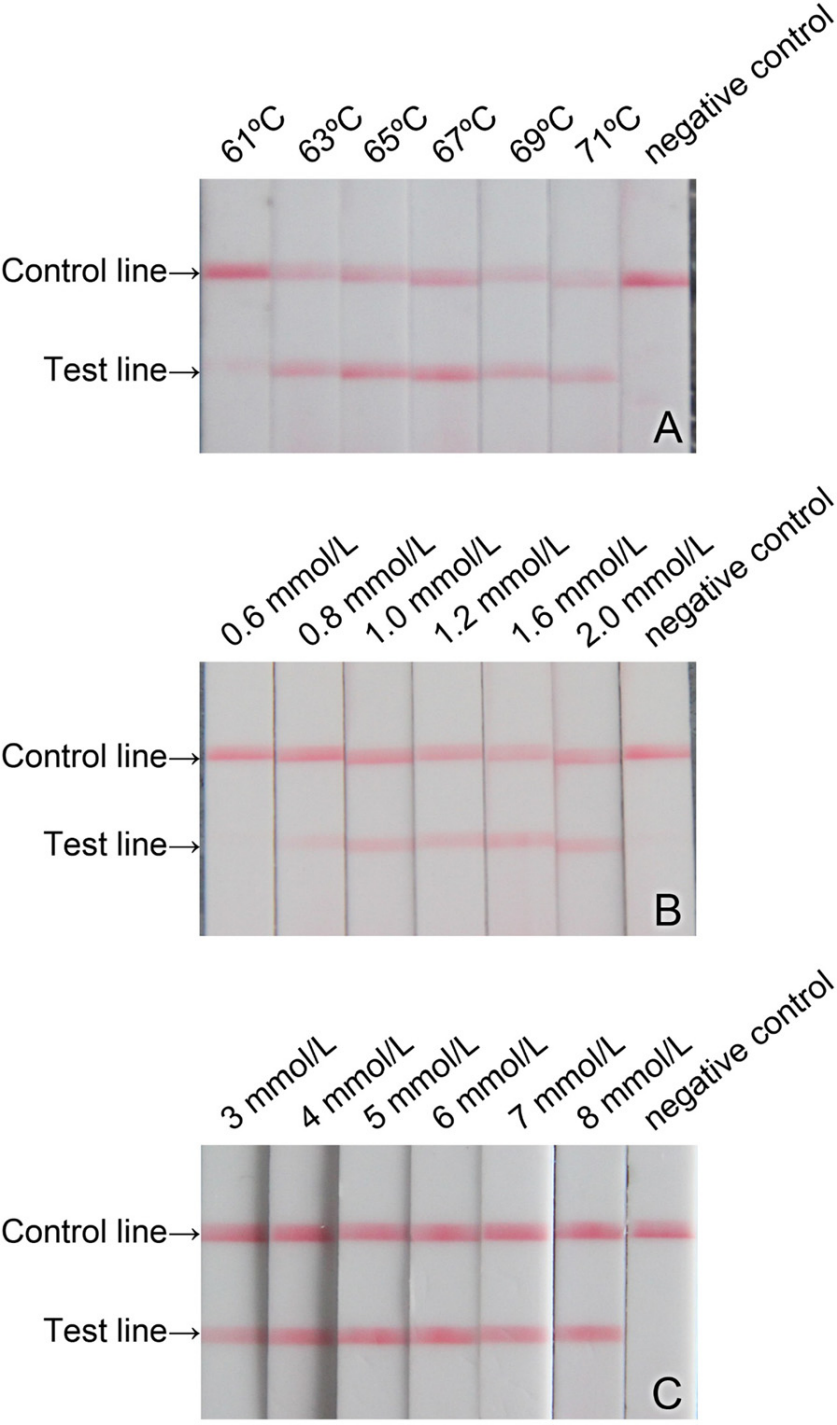
Optimization of CPA-LFD for RGNNV detection. **a** Amplification temperature; **b** Concentration of dNTPs; **c** Concentration of Mg^2+^

In this study, we established the CPA-LFD for RGNNV detection and optimized the amplification reaction temperature, the dNTPs concentration and the concentration of Mg^2+^ successively. The obtained optimal amplification reaction temperature was 69 °C. The optimal concentrations of dNTPs and Mg^2+^ were 1.2 mmol/L and 5 mmol/L (Fig. 2). The finally determined optimal amplification reaction system (25 μL) included 5 mmol/L MgCl_2_, 1.2 mol/L Betaine, 1.2 mmol/L dNTPs, 1.6 μmol/L primers 1 s; 0.8 μmol/L primers 2a/3a, 0.2 μmol/L primers 4 s/5a, 2.5 μL of 10 × ThermoPol Buffer, 8 U of *Bst* DNA polymerase, 28 U of M-MLV reverse transcriptase and appropriate amount of RNA template. The reaction system was amplified at 69 °C for 60 min.

### The sensitivity of CPA-LFD method

**Fig. 3:**
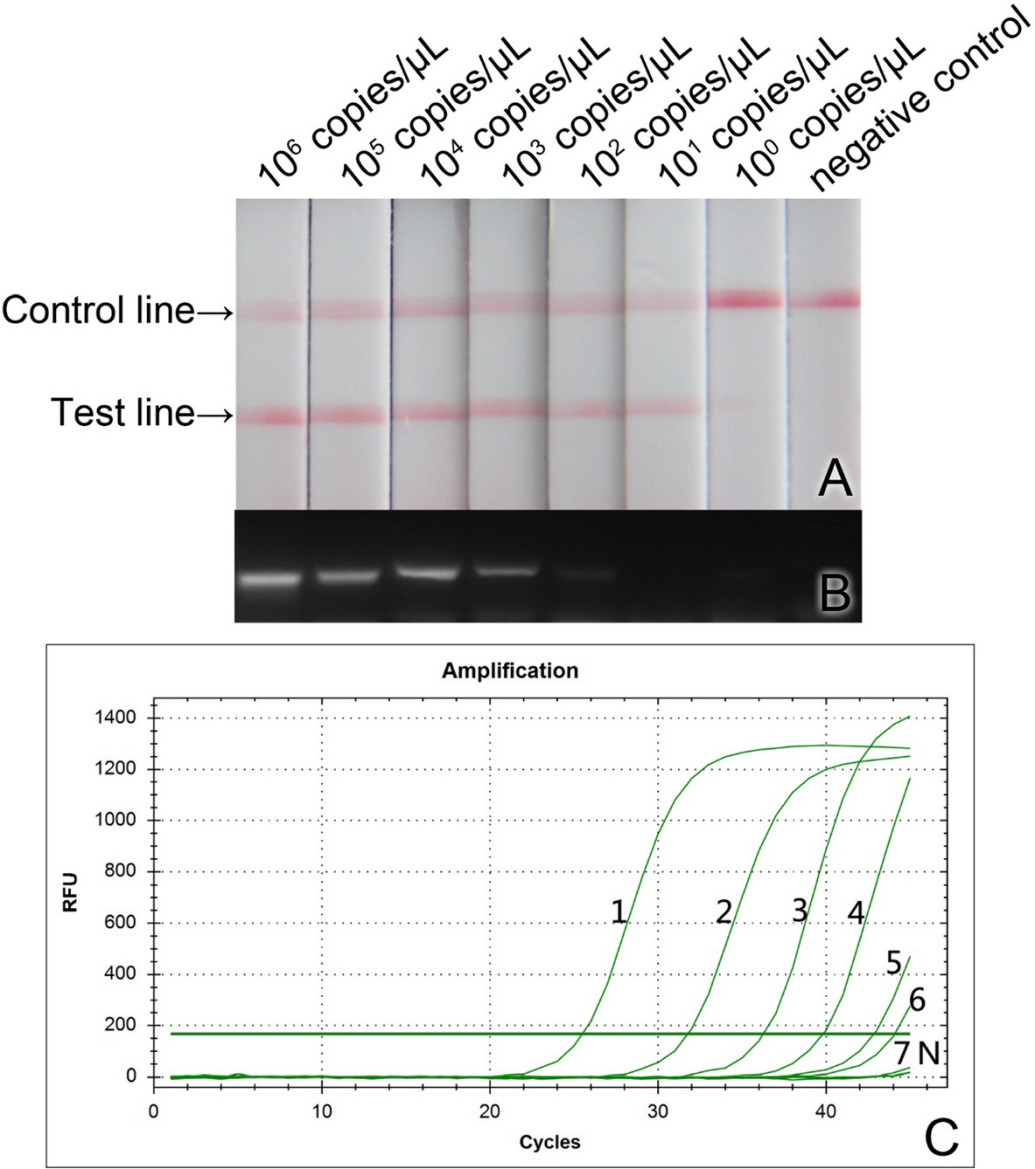
The sensitivity comparison of CPA-LFD, conventional RT-PCR and qRT-PCR. **a** CPA-LFD; **b** conventional RT-PCR; **c** qRT-PCR. The amplification curve 1–7, the concentrations of RGNNV RNA1 standard in the reaction solution was 10^6^, 10^5^, 10^4^, 10^3^, 10^2^, 10^1^, 10^0^ copies/μL, respectively. N: negative control

In the experiments comparing the sensitivities of CPA-LFD, conventional RT-PCR and qRT-PCR methods, the 10-fold series dilutions of RGNNV RNA1 standard solutions were used as the template to conduct nucleic acid amplification with these methods, respectively. The results showed that the lowest limits of detection (LLODs) for CPA-LFD, conventional RT-PCR and qRT-PCR methods were 10^1^ copies/μL, 10^2^ copies/μL and 10^1^ copies/μL, respectively, indicating that the sensitivity of CPA-LFD is 10-times higher than that of the conventional RT-PCR but comparable to that of qRT-PCR (Fig. 3).

### Specificity of CPA-LFD method

**Fig. 4:**
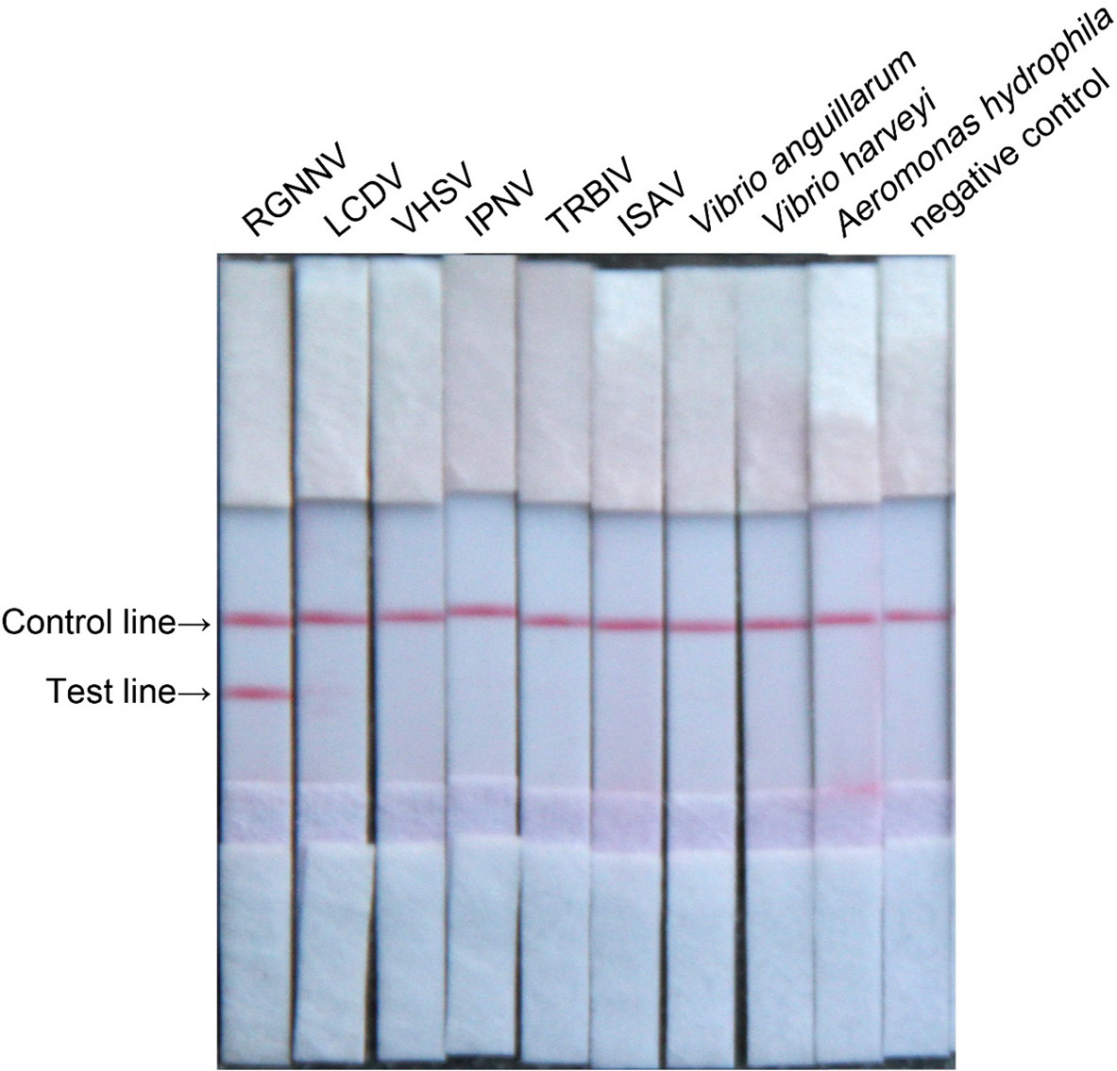
The specificity analysis of CPA-LFD

In this study, RGNNV, VHSV, IPNV, TRBIV, LCDV, ISAV and *V. harveyi, V. anguillarum* and *A. hydrophila* as the template and the RNA prepared from the head of the healthy juveniles of *C. semilaevis* as the negative control were tested with the established CPA-LFD method. The results showed that the RNA of RGNNV was amplified and detected with this method but no any nucleic acid samples from the remaining 8 viruses and bacteria and the RNA from head section of the healthy juveniles of *C. semilaevis* were amplified and detected (Fig. 4). These results indicate that CPA-LFD detection method established by this study has specificity for RGNNV but has no any cross-reactions with other tested fish pathogens. The BLAST results showed that the sequence similarity between our primers and the type species of four NNV genotypes were 98 % (RGNNV), 92 % (SJNNV), 0 % (BFNNV and TPNNV), respectively.

### Application of the established CPA-LFD for RGNNV detection in fish samples

**Fig. 5:**
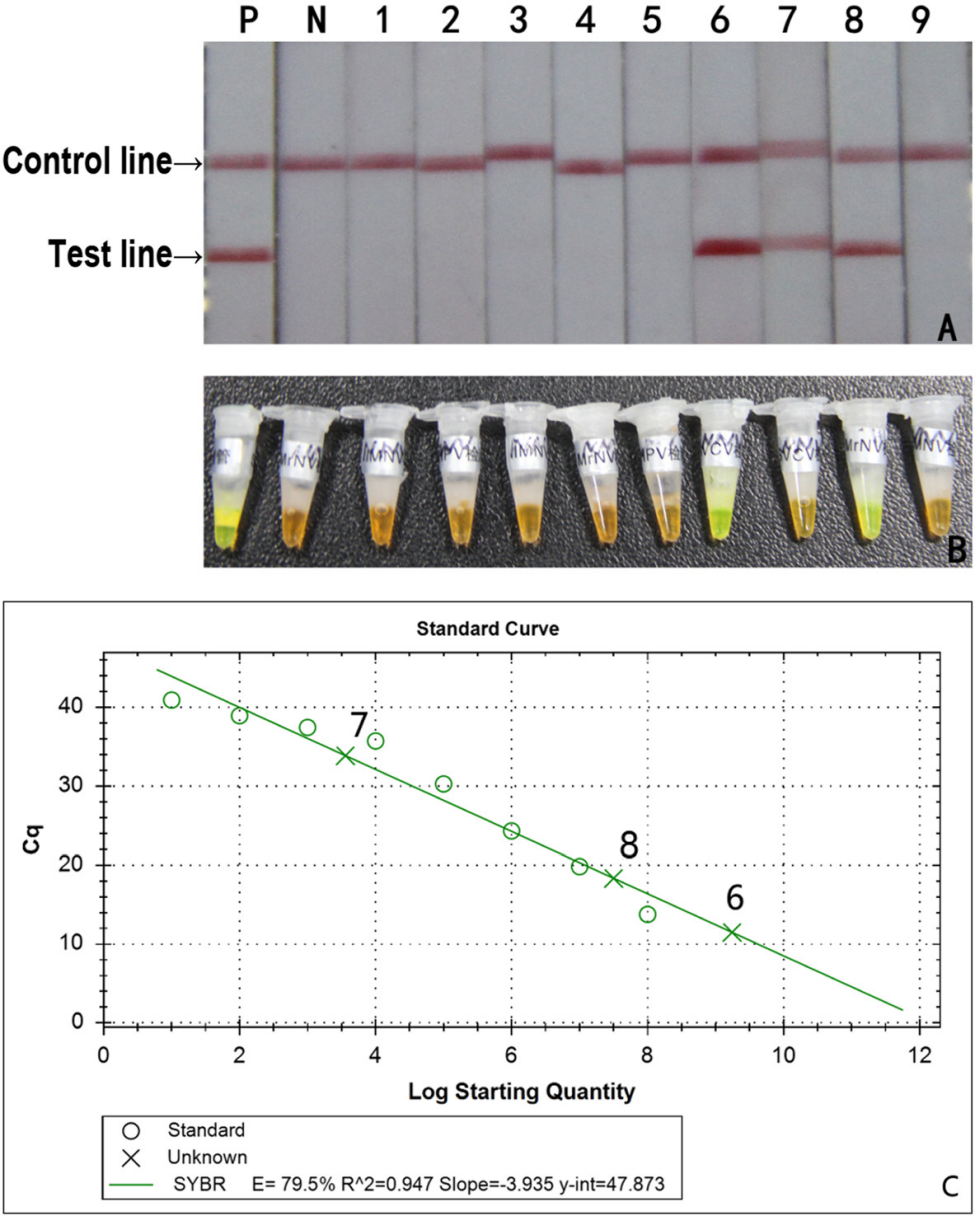
Comparison of CPA-LFD, LAMP kit and qRT-PCR for detection of fish samples. **a** CPA-LFD; **b** LAMP Kit; **c** qRT-PCR. P: positive control. N: negative control. 1–9: fish samples

In this experiment, CPA-LFD, LAMP kit and qRT-PCR were applied to detect for RGNNV in 9 fish samples collected from Shandong province whose infection status was unknown. The results detected with three methods were basically in agreement, i.e. samples 1–5 and 9 were negative for RGNNV while samples 6 and 8 were strongly positive for RGNNV. But for sample 7, the detection result was clearly weak positive detected with CPA-LFD and qRT-PCR whereas it was hard to distinguish whether result detected with LAMP was negative or weakly positive (Fig. 5). Thus, the results detected with CPA-LFD method appear to be more accurate and easier to read.

## Discussion

Red-spotted grouper nervous necrosis virus (RGNNV) is a virus commonly seen during the aquaculture of sea water fishes. This virus mainly infects larvae and juveniles and causes harmful damage to them. The cumulative mortality of infected fish can be as high as 100 %. Its infection causes relatively large economic loss for aquaculture industry. Thus, it is essential to have the availability of an effective method for rapid and accurate detection of RGNNV to prevent and control the fish diseases caused.

Compared to PCR-based detection technologies, CPA and LAMP do not need the thermal denature and the changes in temperature and thus, do not need the complicated and precise equipment and the time for detection is short. However, LAMP required an initial denaturation step but CPA does not. Thus, CPA is simpler, quicker, cheaper and more stable than LAMP [14]. The use of LFD to analyze the CPA-amplified results only took 10 min to observe the results while agarose gel electrophoresis usually took about 40 min. LFD does not need to use the nucleic acid-staining dyes such as GeneFinder and ethidium bromide which can cause harmful effects on human. Owning to these advantages mentioned above, LFD has been widely applied in the detection of various pathogens [16, 24, 25]. Its sensitivity is comparable to or slightly higher than that of agarose gel electrophoresis [22, 24]. The CPA-LFD method has been applied in the *in vitro* detection and diagnosis of human pathogens including *Mycobacterium tuberculosis* [15], *Enterobacter sakazakii* [16], and thrombocytopenia syndrome virus [20] etc. However, there have been few reports about its application in the detection of pathogens that cause fish diseases. In this study, we established a combination of CPA with lateral flow dipstick (LFD) and applied it to detect RGNNV in fish samples.

In this study, by comparing the sensitivities of CPA-LFD, conventional RT-PCR and qRT-PCR, we found that the LLODs for CPA-LFD was 10^1^ copies/μL, which is 10 times higher than that of the conventional RT-PCR and is comparable to that of qRT-PCR. CPA-LFD was a detection method with high sensitivity when it was applied in the transgenic detection, its sensitivity was also 10^1^ copies/μL [23].

The specificity of the CPA-LFD assay was also performed, which revealed the CPA-LFD assay was specific to RGNNV without any cross-reaction with other tested viruses and bacteria. The BLAST result showed that the sequence similarity between our primers and SJNNV was very high (92 %) and it means the CPA-LFD can detect SJNNV potentially. In contrast, the CPA-LFD assay has no cross-reaction with other two NNV genotypes (BFNNV and TPNNV) because the sequence similarities were 0 %. Similar results in LAMP were also reported by Rungkarn et al. [11].

With the CPA-LFD method established in this study, the amplification reaction took place at a constant temperature of 69 °C and the results were visualized by naked eyes via a simple LFD. The entire reaction process only required pieces of simple equipment for maintaining the constant temperature but did not need the large and complex instrument. With CPA-LFD method, the amplification time by CPA was 1 h and the entire procedures from preparation of samples to acquirement of detection results needed only about 1.5 h while the conventional RT-PCR required 2–3 h and nested RT-PCR required longer time [8]. It has been indicated that CPA isothermal reaction for 45 min, the amplified product could be obviously visualized [15]. In this study, we did not test whether the amplification time could be further shortened.

A previous study established a LAMP method and found that its sensitivity for RGNNV could reach 10^1^ copies/μL [9]. The results detected with CPA-LFD method were basically in line with those detected with LAMP kit. However, LAMP needs to use dyes for development color and it is hard to clearly distinguish the weakly positive results from the negative results with naked eyes and erroneous judgment can be easily made [21]. With LFD, the weakly positive results and the negative results can be more obviously visualized and compared and the erroneous judgment can be reduced to certain extend.

## Conclusions

In summary, in this study, we established a CPA-LFD method specific for the detection of RGNNV. This convenient method possesses the characteristics of shorter detection time, high sensitivity and strong specificity and thus can have an application potential not only suitable for the use in laboratories but is more suitable for the aquaculture farms for rapid detection of this virus on the pot.
